# L-Arginine Stimulates Fibroblast Proliferation through the GPRC6A-ERK1/2 and PI3K/Akt Pathway

**DOI:** 10.1371/journal.pone.0092168

**Published:** 2014-03-20

**Authors:** Takashi Fujiwara, Shigeyuki Kanazawa, Ryoko Ichibori, Tomoko Tanigawa, Takuya Magome, Kenta Shingaki, Shingo Miyata, Masaya Tohyama, Ko Hosokawa

**Affiliations:** 1 Department of Plastic Surgery, Osaka University Graduate School of Medicine, Suita-shi, Osaka, Japan; 2 Department of Child Development and Molecular Brain Science, United Graduate School of Child Development, Osaka University, Suita-shi, Osaka, Japan; 3 Department of Research & Development Noevir Co., Ltd. Higashiomi, Shiga, Japan; 4 Division of Molecular Brain Science, Research Institute of Traditional Asian Medicine, Kinki University, Osakasayama, Osaka, Japan; Yokohama City University School of Medicine, Japan

## Abstract

l-Arginine is considered a conditionally essential amino acid and has been shown to enhance wound healing. However, the molecular mechanisms through which arginine stimulates cutaneous wound repair remain unknown. Here, we evaluated the effects of arginine supplementation on fibroblast proliferation, which is a key process required for new tissue formation. We also sought to elucidate the signaling pathways involved in mediating the effects of arginine on fibroblasts by evaluation of extracellular signal-related kinase (ERK) 1/2 activation, which is important for cell growth, survival, and differentiation. Our data demonstrated that addition of 6 mM arginine significantly enhanced fibroblast proliferation, while arginine deprivation increased apoptosis, as observed by enhanced DNA fragmentation. In vitro kinase assays demonstrated that arginine supplementation activated ERK1/2, Akt, PKA and its downstream target, cAMP response element binding protein (CREB). Moreover, knockdown of GPRC6A using siRNA blocked fibroblast proliferation and decreased phosphorylation of ERK1/2, Akt and CREB. The present experiments demonstrated a critical role for the GPRC6A-ERK1/2 and PI3K/Akt signaling pathway in arginine-mediated fibroblast survival. Our findings provide novel mechanistic insights into the positive effects of arginine on wound healing.

## Introduction

l-Arginine, traditionally classified as a nonessential amino acid, is now considered conditionally essential for tissue healing and survival [1], [2]. In normal conditions, l-arginine is produced endogenously according to the needs of the tissues/cells; however, endogenous synthesis of l-arginine may be insufficient during metabolic stress, organ maturation, and development [3]. A number of studies have demonstrated that arginine supplementation is also important in wound healing. The beneficial effects of l-arginine on wound healing are mediated by nitric oxide (NO), which is synthesized from l-arginine through the action of nitric oxide synthase during the wound healing process. In fibroblasts, NO supports collagen synthesis, which is essential for scar formation [4], [5]. However, little is known about the molecular mechanisms mediating this process. Moreover, while l-arginine has been shown to stimulate proliferation in intestinal cells and trophectoderm cells [6], [7], the effects of l-arginine treatment on fibroblast proliferation have not been reported.

Wound healing involves a cascade of events, including blood clotting, inflammation, new tissue formation, and tissue remodeling [8]. This complex process requires the collaborative efforts of many types of cells: immune cells, endothelial cells, keratinocytes, and fibroblasts [9]. Fibroblast migration and proliferation within the wound site play a key role in the formation of granulation tissue. Fibroblasts migrate into the wound tissue, where they proliferate and deposit extracellular matrix. Various intracellular and intercellular pathways are activated and coordinated to facilitate these processes of fibroblast migration and proliferation [10]–[12]. l-arginine has been shown to enhance cell migration via the phosphoinositol 3-kinase/mammalian target of rapamycin pathway in enterocytes [13]. Moreover, a number of mammalian target of rapamycin pathway kinases interact with the actin cytoskeleton in advancing lamellipodia and regulate fibroblast migration [14]. However, the effects of l-arginine supplementation on fibroblast proliferation have not been clearly elucidated.

Members of the mitogen-activated protein kinase (MAPK) family represent important mediators of signal transduction pathways and are required to facilitate the effects of growth factors and other proteins. The pathway mediated by Ras-dependent extracellular signal-regulated kinase (ERK) 1/2, the prototypical MAPK, is one of the most frequently studied signaling systems; ERK1/2 signaling is known to control the expression of various cell-cycle regulators and to participate in multiple cellular functions, such as proliferation, differentiation, and apoptosis [15]–[17].

Recently some studies implicated that the serine threonine kinase Akt/PKB blocks cellular apoptosis and promote cell survival in response to growth factor induction [18], [19]. Furthermore, PI3K and MAPK activation by basic amino acids such as l-lysine, l-arginine and l-ornithine is known be activated by GPRC6A which is a member of the G protein-coupled receptor 3 family. However, the signaling pathway of fibroblast cellular function by l-arginine through activation of GPRC6A receptor is not well elucidated [20], [21].

In the present study, we investigated the effects of l-arginine on fibroblast proliferation and examined the involvement of the GPRC6A, Akt, ERK1/2 pathway and its downstream target, cAMP response element binding protein (CREB), in fibroblast proliferation and survival.

## Results

### L-Arginine Stimulated Fibroblast Proliferation

**Figure 1:**
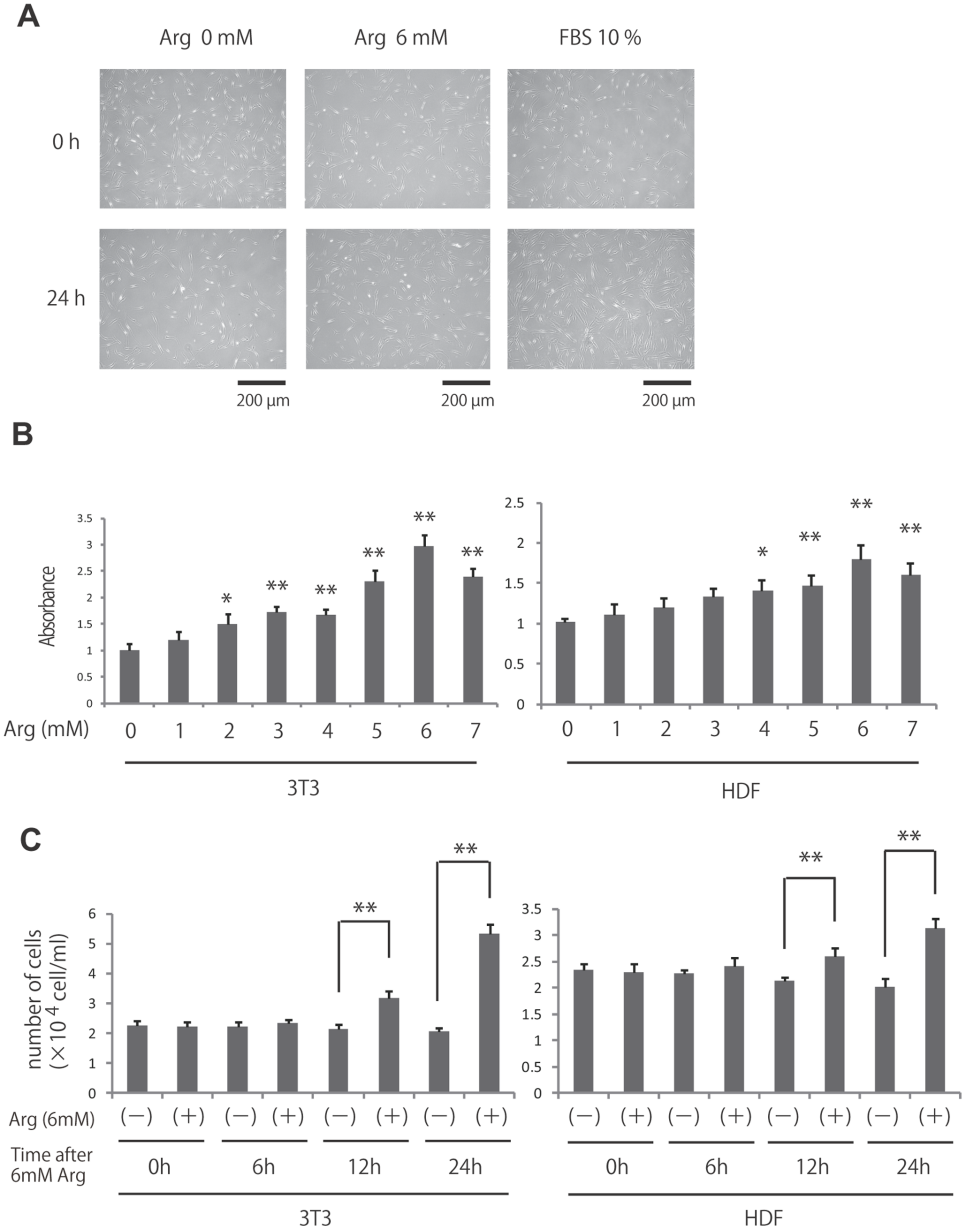
Effects of l-arginine on fibroblast proliferation. (A) This photograph shows human dermal fibroblast at 24 h after treatment with or without 6 mM l-arginine. DMEM with 10%FBS was used as positive control. (B) Fibroblast proliferation was determined by MTS assay after treatment with the indicated concentrations of l-arginine (0–7 mM). Results are expressed as the mean ± SEM of 3 independent experiments. *p<0.05, **p<0.01, as compared with the control group (Student’s t test). (C) The number of trypan blue negative fibroblast at 0, 6, 12 and 24 h after treated with 0 or 6 mM l-arginine. **p<0.01, as compared with the control group (Student’s t test).

l-Arginine has been shown to stimulate the proliferation of intestinal cells and trophectoderm cells [6], [7]; however, the effects of l-arginine treatment on fibroblast proliferation have not been reported. Therefore, we first examined whether treatment with l-arginine affected fibroblast proliferation (Fig. 1A, B, C). Following 24 h of l-arginine starvation, NIH3T3 and primary human dermal fibroblasts (HDF) were treated with various concentrations of l-arginine (0–7 mM) for 24 h, and cell viability was then assessed. Significant dose-dependent proliferation was observed at l-arginine concentrations above 2 mM with NIH3T3 and 4 mM with HDF. Six millimolar l-arginine produced maximum stimulation of proliferation, inducing a 3.0-fold increase in NIH3T3 fibroblast proliferation and 1.8-fold increase in HDF compared to cells deprived of l-arginine. In order to evaluate cell growth and survival of NIH3T3 and HDF with or without l-arginine treatment, we performed trypan-blue dye exclusion cell counting at 0, 6, 12, and 24 h post culture. The results demonstrated trypan-blue excluded cell increase at 12, 24 h in the group with l-arginine cultured NIH3T3 and HDF without decreasing the trypan-blue positive cells (Fig. 1C). Thus, these data demonstrated that l-Arginine induced fibroblast proliferation.

### L-Arginine Decreased Apoptosis in Fibroblasts

**Figure 2:**
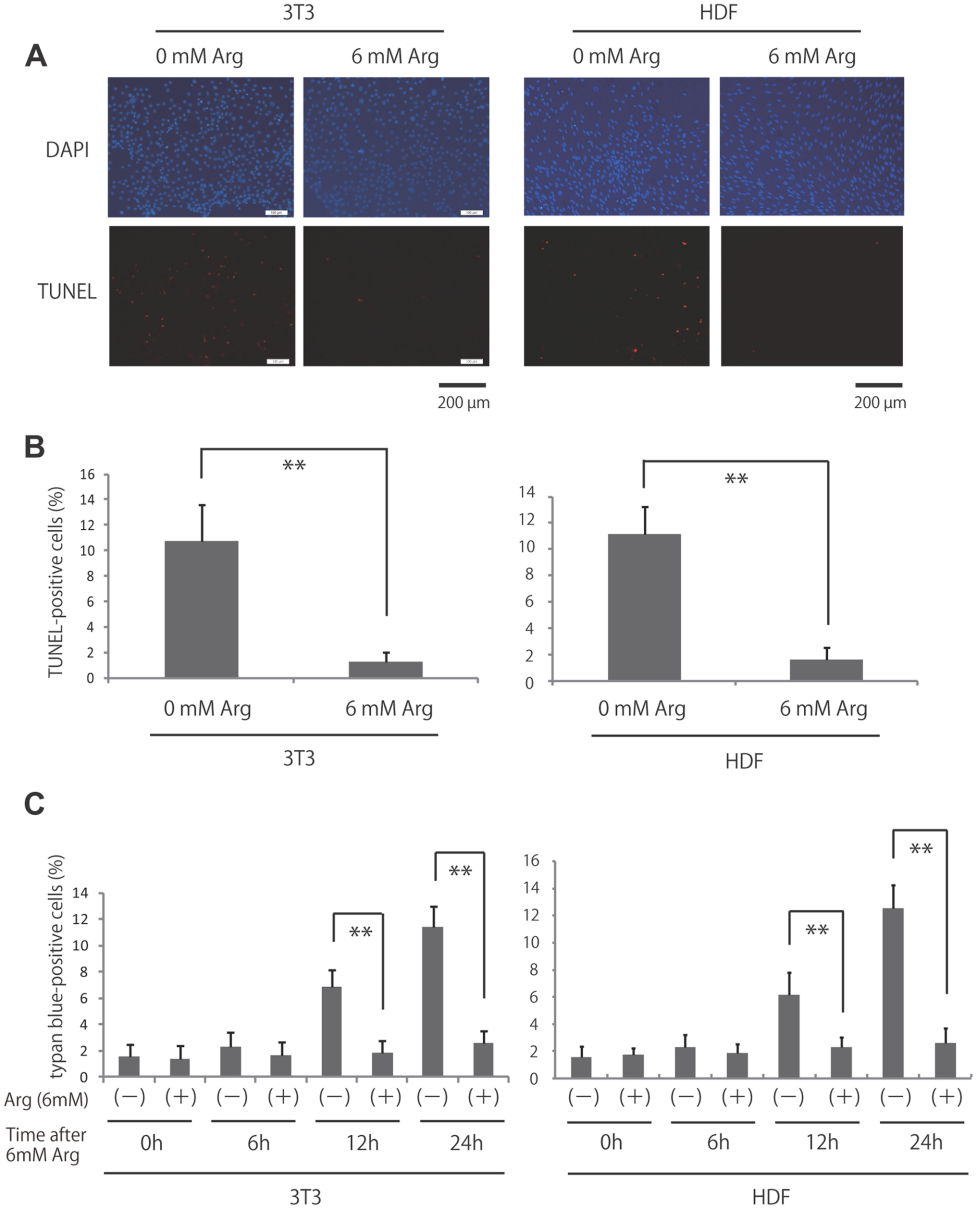
Effects of l-arginine deprivation and stimulation on apoptosis in fibroblasts. (A) Apoptosis was measured using the TUNEL assay in l-arginine-deprived and -treated fibroblasts. (B) Quantification of the data from (A). **p<0.01, as compared with the control group (Student’s t test). (C) The number of cell death using the 0.1% trypan blue staining in l-arginine-deprived and -treated fibroblasts. **p<0.01, as compared with the control group (Student’s t test).

Normal cells require growth factors for proliferation and survival. Growth factor deprivation can lead to apoptosis [22]. Therefore, we next determined whether l-arginine played an important role in preventing apoptosis by examining DNA fragmentation as a marker of apoptosis in the presence or absence of arginine (Fig. 2A, B). l-Arginine-deprived NIH3T3 and HDF demonstrated a significant increase in the percentage of terminal deoxynucleotidyltransferase-mediated dUTP-biotin nick end labeling (TUNEL)-positive cells compared with cells maintained in 6 mM arginine (NIH3T3; 10.7% ±2.9% vs. 1.3% ±0.8%, HDF; 11.1% ±2.1% vs. 1.6% ±0.9% respectively). Furthermore, we performed trypan-blue dye at 0, 6, 12, and 24 h after l-arginine treatment. After the treatment, NH3T3 and HDF both demonstrated significant decrease in trypan-bule positive cells (NIH3T3; 11.4% ±1.5% vs. 2.6% ±1.0%, HDF; 12.6% ±1.7% vs. 2.6% ±1.1% respectively at 24 h) (Fig. 2C) Thus, our data demonstrated that l-arginine decreased apoptosis in fibroblasts. In conclusion, the results suggest that l-arginine inhibits apoptosis.

### L-Arginine Activated ERK1/2, PI3K-Akt, cAMP-PKA and CREB

**Figure 3:**
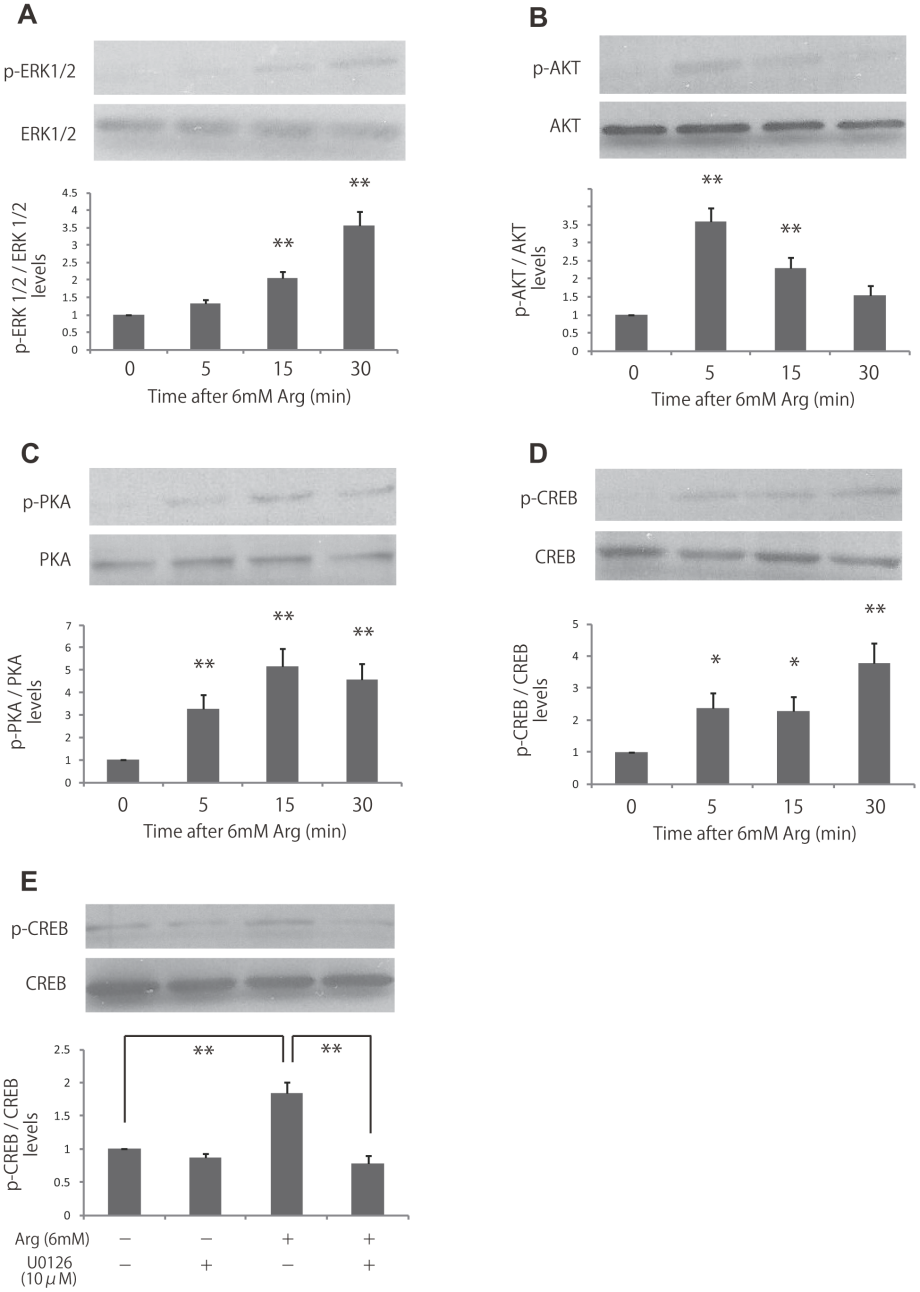
Effects of l-arginine stimulation on the activities of ERK, Akt, PKA and CREB. (A, B, C, D) ERK, Akt, PKA and CREB activities were analyzed by immunoblotting at 5, 15, and 30 min after stimulation with 6 mM l-arginine. Densitometry measurements for p-ERK, p-Akt, p-PKA and p-CREB were normalized to the amount of total ERK, Akt, PKA and CREB, respectively. (E) Fibroblasts were treated with 10 μM U0126, and then cells were stimulated with 6 mM l-arginine. The activities of CREB were analyzed by immunoblotting. Results are presented as the fold change compared with untreated cells. Data are expressed as the mean ± SEM of 3 independent experiments (*p<0.05, **p<0.01, Tukey’s post hoc test).

To examine the molecular mechanisms responsible for the proliferative effects of l-arginine in fibroblasts, we evaluated arginine-dependent activation of the ERK1/2, PI3K/Akt and cAMP-PKA pathway, which plays a major role in regulating cell growth, survival, and differentiation. HDF were deprived of l-arginine for 24 h and then supplemented with 6 mM l-arginine for different times (0–30 min). We found that l-arginine supplementation significantly increased levels of phosphorylated ERK1/2, Akt and PKA at 5–15 min (Fig. 3A, B and C).

Since phosphorylated ERK1/2 is known to phosphorylate transcription factors, such as CREB, which regulates the transcription of genes involved in cellular metabolism, growth, migration, and proliferation, we next examined the effects of arginine on CREB phosphorylation. Similar to the effects observed for ERK1/2, CREB phosphorylation was also significantly increased at 5, 15, and 30 min with l-arginine (2.4, 2.3, and 3.8-fold increases, respectively, compared to cells deprived of arginine; Fig. 3D). To confirm the involvement of ERK1/2 in l-arginine induced CREB phosphorylation, we inhibited ERK1/2 by U-0126, a selective MEK inhibitor. The results showed that l-arginine-induced CREB phosphorylation significantly decreased by 68% with ERK1/2 inhibition (Fig. 3E). Taken together, these data showed that l-arginine activated both ERK1/2 and its downstream effector, CREB.

### L-Arginine-induced Fibroblast Proliferation was Blocked by GPRC6A Knockdown

**Figure 4:**
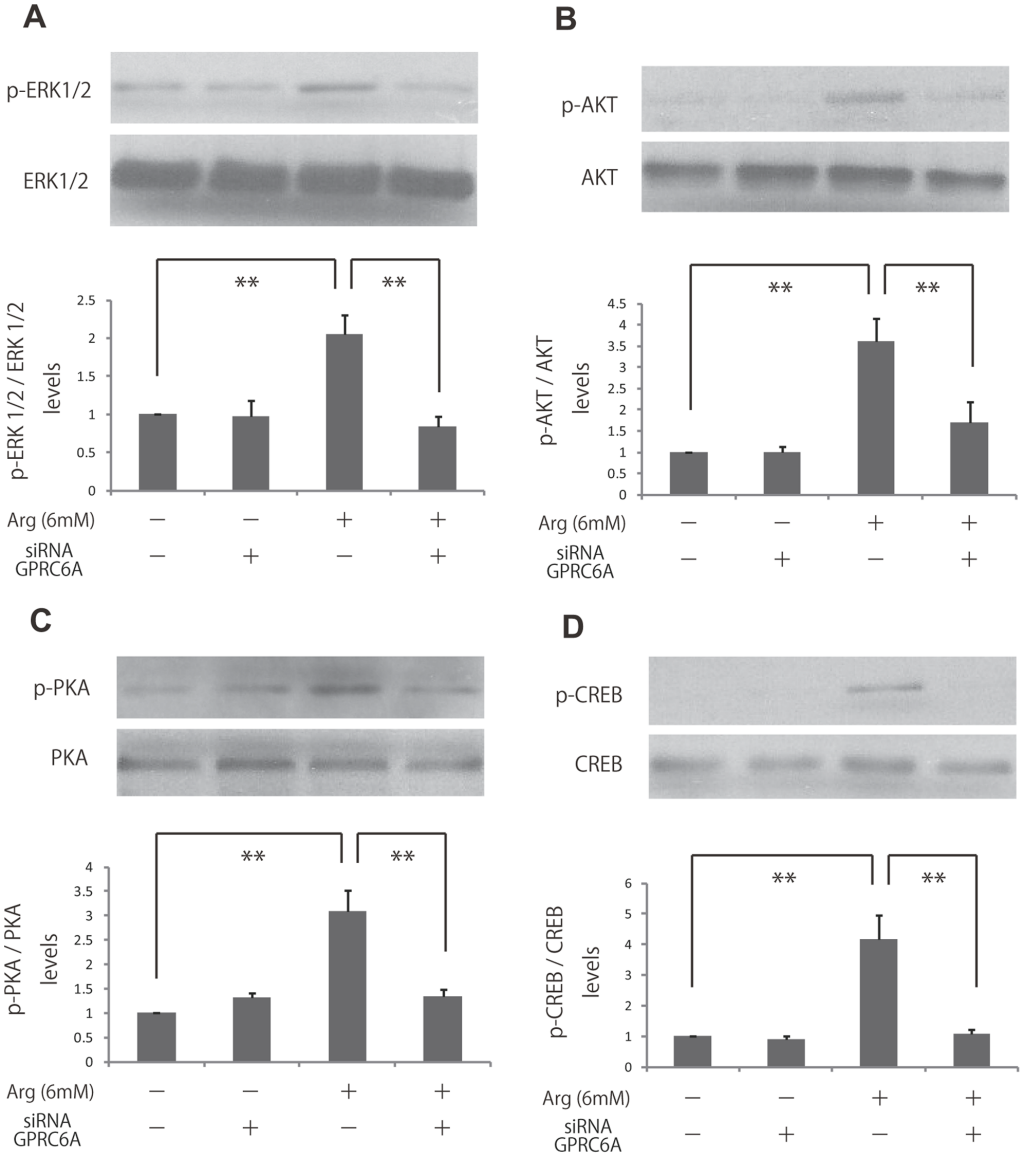
Effects of knockdown of GPRC6A on the activities of ERK, Akt, PKA and CREB in l-arginine-treated fibroblasts. (A, B, C, D) Fibroblasts were treated with si RNA GPRC6A and then cells were stimulated with 6 mM l-arginine. The activities of ERK, Akt, PKA and CREB were analyzed by immunoblotting. Densitometry measurements for p-ERK, p-Akt, p-PKA and p-CREB were normalized to the amount of total ERK, Akt, PKA, and CREB, respectively. Results are presented as the fold change compared with the control group. Data are expressed as the mean ± SEM of 3 independent experiments (**p<0.01, Tukey’s post hoc test).

Since GPRC6A is known to be a receptor for amino acids for basic amino acids such as l-lysine, l-arginine and l-ornithine, we speculated that GPRC6A would be stimulated by l-arginine to activate ERK1/2, Akt, PKA, and CREB. Therefore, we used siRNA to knockdown GPRC6A in HDF to investigate the activation of ERK1/2, Akt, PKA, and CREB. As a result, activation of ERK1/2, Akt, PKA, and CREB were all inhibited due to the knockdown of GPRC6A (69%, 64%, 67%, 74% respectively)(Fig. 4 A, B, C and D). In conclusion, we believe that GPRC6A resides in the upper stream of ERK1/2, Akt, PKA, and CREB to function as the receptor of l-arginine to regulate the cellular signaling.

**Figure 5:**
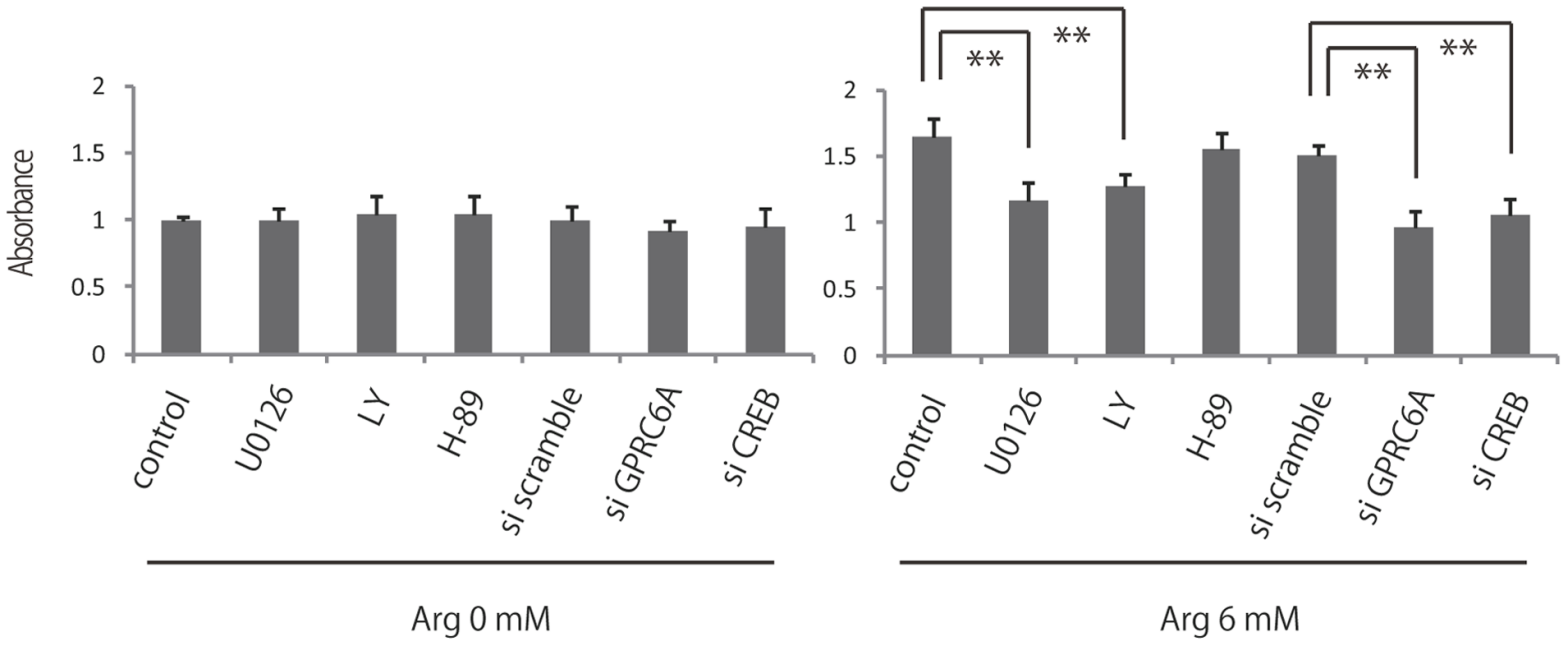
Effects of inhibition of ERK, Akt, PKA, CREB and GPRC6A on the proliferation of l-arginine-treated fibroblasts. Fibroblast proliferation was measured by MTS assay after pretreatment with 0 or 6l-arginine and subsequent treatment with 10 μM U0126, LY294002, H-89, siRNA CREB and siRNA GPRC6A. Results are expressed as the mean ± SEM of 3 independent experiments.

In order to confirm the involvement of GPRC6A, ERK1/2, PI3K-Akt, c-AMP-PKA, and CREB activation in cellular proliferation by l-arginine, we examined the l-arginine-induced fibroblast proliferation by MTS assay using its inhibitors (U-0126, LY294002, H-89 respectively) and siRNA GPRC6A or CREB. The results demonstrated significant cell death or low cell count due to the inhibition of GPRC6A, ERK1/2, PI3K-AKT, and CREB (65%, 70%, 77%, 70% respectively) (Fig. 5). On the other hand, inhibition of PKA did not block l-arginine-induced fibroblast proliferation.

Thus, our data demonstrated that inhibition of GPRC6A, ERK1/2, Akt and CREB blocked l-arginine-induced fibroblast proliferation, thereby suggesting that l-arginine stimulated fibroblast proliferation via GPRC6A, ERK1/2 and PI3K/Akt signaling pathway.

## Discussion

Supplemental l-arginine has been shown to improve wound healing, as manifested by enhanced T-cell-mediated immune function [23], [24], increased wound breaking strength, and collagen deposition [2], [25]. Arginine treatment has also been shown to regulate the cytokine environment at the wound site, reducing tissue interleukin-6 levels in the wound [26]. Thus, l-arginine decreases the adverse effects of increased inflammation in the wound. However, despite the great number of studies demonstrating the beneficial effects of arginine on wound healing, the molecular mechanisms through which it mediates these effects have not been well investigated. Moreover, the effects of l-arginine on fibroblasts, important cells in the wound healing process that are responsible for collagen synthesis in granulation tissue have not been clearly elucidated.

In our present study, we demonstrated that l-arginine supplementation stimulates fibroblast proliferation and that deprivation of l-arginine facilitates fibroblast apoptosis, suggesting that arginine possesses fibroblast proliferation promoting and anti-apoptotic effects. Consistent with this, arginine has been shown to induce proliferation in endothelial cells, intestinal cells and trophectoderm cells [6], [7]. Although we did not show the in vivo role of l-arginine in wound healing, we believe that our data can support the idea of l-arginine taking a great part in wound healing by promoting fibroblast cellular function. To support our findings, Yatabe et al demonstrated the efficacy of clinical application of l-arginine as ARGINAID WATER for accelerating wound healing in pressure ulcer patients [27]. Since the concentration of l-arginine used for our study is much higher compare to clinical use, we assume that l-arginine can function with much lower concentration in vivo. For future study, we plan to investigate the in vivo function of l-arginine in murine or human individuals.

MAPK signaling plays an important role in complex cellular processes, such as proliferation, differentiation, development, transformation, and apoptosis. At least 3 MAPK families have been characterized: ERK, Jun kinase, and p38 MAPK. MAPKs, particularly ERK1/2, play an important role in proliferation, differentiation, and survival processes [15]–[17]. Phosphorylation of ERK1/2 has been linked to activation of CREB [28], [29]; phosphorylated ERK1/2 translocates to the nucleus where it phosphorylates CREB, a transcription factor, which activates genes involved in cell proliferation. In the present study, we demonstrated that l-arginine treatment following a period of starvation resulted in the activation of ERK1/2 and CREB, suggesting the involvement of the entire ERK1/2 pathway in the response to l-arginine in human dermal fibroblasts.

Furthermore, PI3K-Akt is also an important signaling factor for cell survival other than MAPK signaling. At the same time, serine threonine kinase Akt/PKB also acts as an important mediator of metabolic factors and promotes cell survival against several apoptotic stimuli [30]. In our study, we were able to demonstrate that l-arginine induces the activation of Akt signaling and promotes fibroblast proliferation and cell survival. We supported these findings and proved that Akt is an important factor for l-arginine-induced fibroblast cell activity by showing that the activity of l-arginine is inhibited after Akt signaling inhibition.

Moreover, we investigated whether l-arginine stimulates GPRC6A which is known to be the receptor for amino acids such as L-lysine, L-arginine, L-ornithine to activate the ERK and Akt signaling pathway. We were able to prove that GPRC6A is greatly involved in activation of ERK1/2, Akt, PKA, and CREB factors with l-arginine stimulus by showing that GPRC6A knockdown inhibits ERK1/2, Akt, PKA, and CREB activation. In addition, we demonstrated that l-arginine-induced fibroblast proliferation significantly decreases with inhibition of GPRC6A, CREB, ERK1/2 and Akt but was not affected with inhibition of PKA. Therefore, we believe that PKA is not strongly involved in the activation of fibroblast proliferation. All to together, our findings suggest that fibroblast cell proliferation and anti-apoptotic effect is stimulated by l-arginine through the activation of GPRC6A, ERK1/2-CREB and PI3K/Akt pathway.

The effects of arginine supplementation may be mediated by NO synthesis. l-Arginine has been identified as a substrate for the production of the biologic effector molecule NO by a group of isoenzymes termed nitric oxygen synthases. This pathway is present in many tissues and cells, including fibroblasts. NO is a highly diffusible intercellular signaling molecule that regulates the activity of several transcription factors. NO has been shown to increase proliferation in BALB/c 3T3 fibroblasts and endothelial cells [31], [32]. Studies have also identified G proteins, and particularly p21^ras^, as central targets of NO [33], [34]. The mechanism of p21^ras^ activation is due to S-nitrosylation of a cysteine residue, which promotes guanine nucleotide exchange [34]. In addition, NO has been shown to signal downstream of p21^ras^ by activation of the ERK pathway in human T cells [35]. This ERK pathway is typically activated by growth factors via a p21^ras^-dependent signal transduction pathway [36], [37]. Therefore, it is possible that l-arginine-induced fibroblast proliferation and ERK1/2 activation may be attributed to the synthesis of NO by nitric oxygen synthase.

In conclusion, the present experiments demonstrated a critical role for activation of GPRC6A along with the activation of ERK1/2-CREB and PI3K/Akt pathway in l-arginine-mediated fibroblast proliferation and survival. Our findings provide novel mechanistic insights into the positive effects of l-arginine on fibroblast proliferation and survival.

## Materials and Methods

### Cell Culture

Human dermal fibroblast and NIH 3T3 fibroblasts were used for experiments. Cells were cultured in Dulbecco’s modified Eagle medium (DMEM) containing 10% fetal bovine serum, 100 U/mL penicillin, and 100 μg/mL streptomycin in a humidified incubator at 37°C with a 5% CO_2_ atmosphere. l-Arginine starvation was performed by preparing l-arginine free medium using SILAC™ D-MEM (without l-arginine, l-glutamine, l-lysine) added 584 mg/L l-glutamine and 146 mg/l l-lysine. All experiments were performed in triplicate.

### Cell Viability Assay

Cell survival was determined using a CellTiter 96 AQueous One Solution Cell Proliferation Assay (Promega, WI, USA). Fibroblasts were plated at a density of 5000 cells per well in 96-well plates and incubated for 24 h in DMEM containing 10% fetal bovine serum. After l-arginine starvation for 24 h, fibroblasts were treated with ERK, Akt, PKA inhibitor (10 μM U-0126, 10 μM LY294002, 10 μM H-89) for 1 h and then treated with various concentrations of l-arginine (0–7 mM or 6 mM) for 24 h. After l-arginine treatment, 20 μL of One Solution Reagent was added into each well of the 96-well assay plate containing the samples in 100 μL of culture medium. The fibroblasts were incubated for an additional 2 h at 37°C in a humidified, 5% CO_2_ atmosphere. The production of formazan produced by viable cells was measured at an absorbance of 490 nm using a 96-well plate reader.

### Cell Counting with Trypan Blue Dye Exclusion

Fibroblasts were plated on 35 mm dish and incubated for 24 h in DMEM containing 10% FBS. After l-arginine starvation for 24 h the cells were treated with 0 or 6 mM l-arginine. The cells were detached by trypsinization at 0, 6, 12 and 24 h after L-arginine treatment and exposed to 0.1% trypan blue. The number of trypan blue negative cells was counted using a microscopic counting chamber.

### Western Blot Analysis and Immunoprecipitation

Cultured fibroblasts were starved for 24 h in serum-free DMEM without l-arginine. Six millimolar l-arginine was then added, and cells were harvested at specified times (0–30 min). For inhibitor studies, cells were deprived of l-arginine for 24 h and then treated with MEK inhibitor (U-0126 [10 μM]) in the presence or absence of l-arginine. After treatment, cells were lysed in RIPA buffer containing 1 mM Na_3_VO_4_, 1 mM NaF, and Protease Inhibitor Cocktail (Roche Diagnostics, Basel, Switzerland), incubated for 20 min at 4°C, and centrifuged at 15,000×*g* for 15 min at 4°C. The proteins were separated by SDS-PAGE and electrotransferred onto Immobilon-P Transfer Membranes (Millipore Japan, Tokyo, Japan). The membranes were incubated in TBS containing 5% skim milk and 0.05% Tween-20 for 60 min and blotted with primary antibodies at 4°C overnight. The following primary antibodies were used: anti-phospho-ERK1/2 (1∶1000, Cell Signaling Technology, MA, USA), anti-ERK1/2 (1∶1000, Cell Signaling Technology), anti-phospho-CREB (1∶1000, Cell Signaling Technology), anti-CREB (1∶1000, Cell Signaling Technology), anti-phospho-Akt (1∶1000, Cell Signaling Technology), anti-Akt(1∶1000, Cell Signaling Technology), anti-phospho-PKA (1∶1000, Cell Signaling Technology), and anti-PKA (1∶1000, Cell Signaling Technology). The membranes were incubated for 1 h with anti-mouse or anti-rabbit horseradish peroxidase-linked secondary antibodies (1∶2000, Cell Signaling Technology). Reaction products were visualized by detection of chemiluminescence using an ECL Western Blotting Detection System (GE Healthcare, Piscataway, NJ, USA). Quantification of relative band densities was performed by scanning densitometry using Image J software (National Institute of Health, Bethesda, MD, USA).

### TUNEL Apoptosis Assay

Apoptosis was determined by TUNEL assay using an In Situ Cell Death Detection Kit TMR Red (Roche), according to the manufacturer’s instructions. The fibroblasts were maintained in DMEM containing 10% fetal bovine serum for 2 days and cultured in serum-free DMEM without l-arginine for 24 h. Then, the cells treated with or without 6 mM l-arginine for 24 h. The cells were fixed with paraformaldehyde solution (4% in phosphate-buffered saline [pH 7.4]) for 60 min at room temperature and washed with PBS 5 times. Permeabilization was carried out with 0.1% Triton X-100 in PBS for 10 min, followed by incubation in a TUNEL reaction mixture containing terminal deoxynucleotidyltransferase and TMR red-labeled nucleotides for 1 h. The coverslips were mounted onto the slides using VECTASHIELD Mounting Medium with DAPI (Vector Laboratories, Peterborough, England). Fluorescence images were taken using a microscope (model IX-70; Olympus) equipped with a CCD Camera (CoolSNAP HQ; Nippon Roper, Chiba, Japan). One hundred cells per experiment were randomly selected, and the percentage of TUNEL-positive cells was measured.

### RNA Interference Experiments

siRNA duplex for the GPRC6A or CREB gene was synthesized by Invitrogen with help of the D-LUX Designer (Invitrogen Japan, Tokyo, Japan). The GPRC6A siRNA (59-GGGAUGCUGAUUUACUUCAUAGCUU-39) was designed to target coding the region of human GPRC6A mRNA sequence (GenBank accession no. NM_148963). The CREB1 siRNA (59-GCCCAGCAACCAAGUUGUUGUUCAA-39) was designed to target coding the region of human CREB1 mRNA sequence (GenBank accession no. NM_004379). Primary human fibroblasts were transfected with GPRC6A or CREB1 siRNA using Lipofectamine RNAiMAX (Invitrogen Japan) and Opti-MEM (Invitrogen Japan) by the manufacturer’s protocol. The final concentration of siRNA was 10 nM. Stealth RNAi Negative Control Medium GC Duplex (Invitrogen Japan) was used as a control. The transfected cells were used for experiments after 48 h.

### Statistical Analysis

All data shown are expressed as the mean ± SE of the values obtained from 3 individual experiments. Data from each independent experiment were normalized to the control sample in that particular experiment. Differences between results were analyzed by using Student’s t test. Multiple group comparisons were performed using a one-way analysis of variance, followed by the Tukey’s post hoc test. *P*-values of less than 0.05 were considered statistically significant.
